# A comparative study on the efficacy and safety of biosimilar (Rimmyrah) and reference ranibizumab in patients with diabetic macular edema

**DOI:** 10.3389/fphar.2026.1773081

**Published:** 2026-02-10

**Authors:** Gaixia Zhai, Xiaona Ren, Bing Xu, Dongming Mi

**Affiliations:** Zibo Central Hospital, Zibo, China

**Keywords:** diabetic macular edema, foveal avascular zone, ranibizumab biosimilar, reference ranibizumab, vascular density

## Abstract

**Objective:**

This study evaluated the efficacy and safety of the biosimilar Rimmyrah versus the reference ranibizumab in patients with diabetic macular edema (DME).

**Methods:**

This retrospective study included 70 patients with DME. They were divided into two groups: 35 patients (35 eyes) received the reference ranibizumab, and 35 patients (35 eyes) received the biosimilar ranibizumab. All patients were treated following a 3+ pro re nata (PRN) regimen. Best-corrected visual acuity (BCVA) and central retinal thickness (CRT) were compared between the groups at 3, 6, and 12 months post-treatment. Additionally, the foveal avascular zone (FAZ) area and macular vessel density were compared at baseline and 12 months, along with the total number of intravitreal injections required.

**Results:**

There were no statistically significant differences between the two groups in BCVA or CRT at any time point (all P > 0.05). Consistent with the therapeutic effect of ranibizumab, both groups showed significant improvements from baseline in BCVA and CRT (all P < 0.05). Similarly, no intergroup differences were found in FAZ area, superficial vascular density (SVD), or deep vascular density (DVD) at baseline or 12 months (all P > 0.05), with both groups exhibiting significant within-group improvements post-treatment (reduced FAZ, increased SVD and DVD; all P < 0.05). No statistically significant difference was observed in the mean number of intravitreal injections between the reference ranibizumab group (3.43 ± 0.65) and its biosimilar group (3.69 ± 0.76) during the study period (P = 0.1530). No treatment-related serious ocular or systemic adverse events occurred in either group during the 12-month follow-up.

**Conclusion:**

The ranibizumab biosimilar (Rimmyrah) showed similar safety and efficacy profiles to its reference product in the treatment of diabetic macular edema.

## 1 Introduction

Diabetic retinopathy (DR) is a prevalent complication, affecting approximately one-quarter to one-third of all diabetic patients. Notably, according to the International Diabetes Federation’s 2015 global survey, about 5% of these individuals develop diabetic macular edema (DME), a major contributor to blindness in this population (Yau et al., 2012). Currently, it is widely accepted among scholars (Wei et al., 2021; Xia et al., 2024; Feng et al., 2013) that retinal ischemia-hypoxia and the breakdown of the blood-retinal barrier serve as principal contributors to the upregulation of various factors. This upregulation subsequently leads to increased vascular permeability, thereby facilitating the development of macular edema. Accordingly, VEGF antagonist therapy has gained considerable attention as a treatment strategy for DME. The therapeutic effect of these drugs is mediated through VEGF inhibition, resulting in the preservation or enhancement of visual acuity in affected individuals (Schmidt-Erfurth et al., 2014). Ranibizumab (Lucentis) is a recombinant humanized monoclonal IgG1 antibody that targets all isoforms of VEGF-A. Numerous clinical trials have confirmed the significant efficacy of ranibizumab in treating DME (Dervenis et al., 2017; Nikhil et al., 2025).

However, the high cost of the reference ranibizumab places it beyond the financial reach of the majority of patients. This limited affordability may lead to poorer treatment adherence and, consequently, restrict its widespread clinical application. Biosimilars are defined as biological products that are highly similar to an already approved reference biologic and are marketed after the originator’s patent expires, with demonstrated equivalence in safety and efficacy (Sharma et al., 2021). Qilu Pharmaceutical’s biosimilar ranibizumab injection (Rimmyrah) received marketing approval from China’s National Medical Products Administration (NMPA) on 19 August 2024. This approval marks it as the first ranibizumab biosimilar to be authorized for use in China. The active ingredient of a biosimilar is essentially the same as that of its reference drug, although the production process may introduce minor differences (Sameera et al., 2016). To our knowledge, there are no published reports from clinical studies directly comparing the efficacy of reference ranibizumab and the biosimilar (Rimmyrah) in patients with DME.

This study aims to compare the efficacy and safety of the ranibizumab biosimilar (Rimmyrah) with the reference ranibizumab in patients with DME, thereby providing evidence to support the rational clinical use of biosimilars.

## 2 Materials and methods

### 2.1 Study protocol

This study was designed as a retrospective investigation and received approval from the Medical Ethics Committee of Zibo Central Hospital (Approval No.: 2025 Research No. 320). The research was performed in line with the ethical principles outlined in the Declaration of Helsinki. Before treatment initiation, written informed consent was obtained from each patient after a detailed discussion of the potential risks and benefits associated with intravitreal injections.

### 2.2 Patients

This study retrospectively reviewed the clinical data of 70 patients (70 eyes) with DME from the Department of our institution (January 2021 to December 2024). The patients were allocated to two groups: the reference ranibizumab group and the ranibizumab biosimilar group, each consisting of 35 cases (35 eyes). Both groups were treated according to a “3+ pro re nata (PRN)” protocol. Included patients were newly diagnosed with DME and had completed a comprehensive ophthalmic examination. Exclusion criteria were as follows: macular center retinal thickening secondary to epiretinal membrane, vitreomacular traction, or other pathologies; contraindications to the study medications (Ranibizumab); a history of glaucoma or any intraocular surgery; receipt of macular laser photocoagulation or intravitreal anti-VEGF injections within the preceding 3 months, or intraocular/periocular corticosteroids within the preceding 6 months; severe concomitant cardiac, cerebral, or renal dysfunction; loss to follow-up, transfer of care to another facility, or death; HbA1c > 7%.

In order to control for confounding factors and enhance internal validity, this study excluded patients with HbA1c levels exceeding 7%. Consequently, the study population was limited to a subgroup with well-controlled diabetes, which may restrict the generalizability of the findings to the broader real-world DME population, which includes many individuals with suboptimal glycemic control (HbA1c > 7%). Therefore, future studies involving more diverse patient cohorts are needed to validate these conclusions.

### 2.3 Examination and treatment

A full suite of ophthalmic examinations was conducted pre- and post-treatment. The assessments encompassed slit-lamp biomicroscopy, intraocular pressure (IOP) measurement, evaluation of best-corrected visual acuity (BCVA), fundus photography, optical coherence tomography (OCT), and OCT angiography (OCTA) (Carl Zeiss Meditec AG, RTVue XR). Quantitative assessments focused on retinal structure and microvasculature: central retinal thickness (CRT) was derived from OCT, whereas OCTA provided measurements of the foveal avascular zone (FAZ) area, as well as superficial and deep vascular vascular density (SVD and DVD). For OCTA imaging, a 6 × 6 mm scanning area centered on the fovea was used. A predefined image quality criterion (signal strength ≥7) was applied to select scans for quantitative analysis.

The treatment regimen comprised monthly intravitreal injections of either reference ranibizumab (0.5 mg/0.05 mL; Lucentis; Genentech, Inc., South San Francisco, CA, United States) or a ranibizumab biosimilar (Rimmyrah, 0.5 mg/0.05 mL; Qilu Pharmaceutical). All patients were subsequently monitored on a monthly basis for a minimum duration of 12 months. A uniform set of retreatment criteria was applied to all patients following the initial three-loading doses. Retreatment was administered if any of the following conditions occurred: optical coherence tomography (OCT) revealed a central retinal thickness (CRT) of more than 280 μm; or BCVA had declined by 5 or more ETDRS letters since the previous assessment.

Following a routine aseptic protocol, all intravitreal injections were administered in an operating room. Subsequently, a one-week course of tobramycin-dexamethasone eye drops (four times daily) was prescribed to all patients.

### 2.4 Observation parameters

Quantitative assessments of BCVA (logMAR) and CRT were conducted at baseline and then at 3, 6, and 12 months post-injection. In contrast, measurements of glycated hemoglobin (HbA1c), FAZ area, SVD, and DVD were performed only at baseline and the 12-month follow-up. Additionally, the total number of intravitreal injections administered and any adverse reactions were recorded throughout the treatment period.

### 2.5 Statistical analysis

All statistical analyses were performed with GraphPad Prism 9 software, and a p-value <0.05 was defined as statistically significant. Quantitative data are expressed as mean ± standard deviation (SD). Changes in BCVA, CRT, HbA1c, FAZ area, SVD, and DVD from baseline to post-treatment were analyzed using paired t-tests or Wilcoxon signed-rank tests, as appropriate. Intergroup differences were evaluated using independent-samples t-tests or the Mann-Whitney test. Gender distribution was examined for significant differences between the groups with Pearson’s chi-square test.

## 3 Results

### 3.1 Baseline characteristics

This study included 35 patients (35 eyes) in both the reference ranibizumab group (19 males, 16 females) and the biosimilar ranibizumab group (17 males, 18 females), with no significant difference in sex distribution between groups (P = 0.8112). The mean age was 58.00 ± 9.58 years in the reference group and 58.89 ± 9.82 years in the biosimilar group, which was not statistically significant (P = 0.7037). At baseline, the mean HbA1c level was 6.45% ± 0.20% in the reference ranibizumab group and 6.48% ± 0.18% in the biosimilar group, with no statistically significant difference between them (P = 0.3685). At the 12-month follow-up, the mean HbA1c levels were (6.51 ± 0.21)% in the reference ranibizumab group and (6.49 ± 0.21)% in the biosimilar group. No statistically significant difference was observed between the two groups (P = 0.6258). Baseline characteristics are summarized in Table 1.

**TABLE 1:** Baseline characteristics.

Characteristics (mean ± SD)	Reference ranibizumab group (n = 35)	Ranibizumab biosimilar group (n = 35)	P
Age (years)	58.00 ± 9.58	58.89 ± 9.82	0.7037
Gender	19 males, 16 females	17 males, 18 females	0.8112
BCVA (log MAR)	0.57 ± 0.11	0.58 ± 0.12	0.6416
CRT (µm)	424.7 ± 41.85	437.1 ± 40.32	0.2101
IOP (mmHg)	16.26 ± 3.08	15.20 ± 2.66	0.1287
HbA1c (%)	6.45 ± 0.20	6.48 ± 0.18	0.3685

Abbreviations: BCVA, best-corrected visual acuity; IOP, intraocular pressure; CRT, central retinal thickness; HbA1c, glycated hemoglobin.

### 3.2 Comparison of BCVA and CRT between the two groups before and after treatment

**FIGURE 1:**
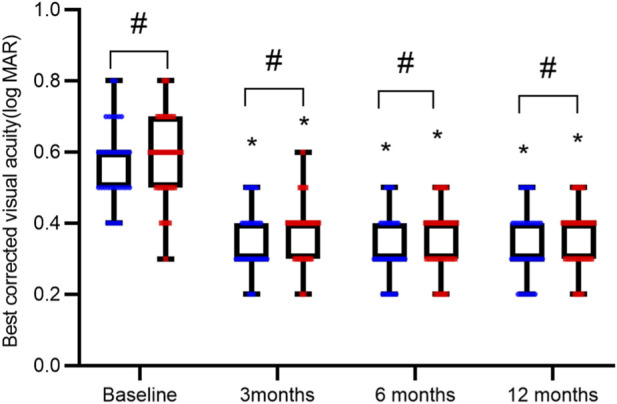
Comparison of Best-Corrected Visual Acuity (BCVA) over Time. BCVA measurements are presented as interleaved box-and-whisker plots for the reference ranibizumab (blue) and ranibizumab biosimilar (Rimmyrah, red) groups (n = 35 per group) at baseline, 3, 6, and 12 months. All individual data points are overlaid. Asterisks (*) denote statistically significant improvement from baseline within the same group (P < 0.05). No significant differences were observed between the two treatment groups at any time point (all P > 0.05).

**FIGURE 2:**
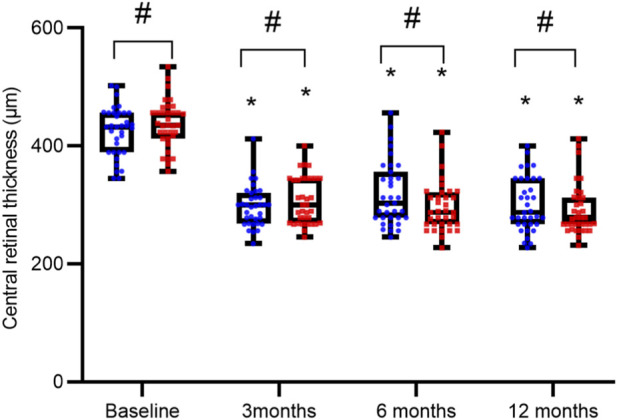
Comparison of Central Retinal Thickness (CRT) over Time. CRT measurements are presented as interleaved box-and-whisker plots for the reference ranibizumab (blue) and ranibizumab biosimilar (Rimmyrah, red) groups (n = 35 per group) at baseline, 3, 6, and 12 months. All individual data points are overlaid. Asterisks (*) denote statistically significant reduction from baseline within the same group (P < 0.05). No significant differences were observed between the two treatment groups at any time point (all P > 0.05).

The two groups exhibited comparable BCVA and CRT levels both at baseline and following treatment, with no intergroup differences reaching statistical significance (all P > 0.05). In contrast, a marked improvement from baseline was observed within each group after intervention, characterized by significantly improved BCVA and reduced CRT (all P < 0.05), as summarized in Table 2 and illustrated in Figures 1, 2.

**TABLE 2:** Comparative changes in BCVA and CRT following intervention.

Group	Indices (mean ± SD)	Baseline	3 months	6 months	12 months
Reference ranibizumab (n = 35)	BCVA (LogMAR)	0.57 ± 0.11	0.35 ± 0.09^*^	0.33 ± 0.09^*^	0.34 ± 0.10^*^
	CRT/μm	424.7 ± 41.85	298.1 ± 34.83^*^	318.4 ± 54.35^*^	300.5 ± 46.39^*^
Ranibizumab biosimilar (n = 35)	BCVA (LogMAR)	0.58 ± 0.12	0.38 ± 0.08^*^	0.35 ± 0.09^*^	0.36 ± 0.09^*^
	CRT/μm	437.1 ± 40.32	306.6 ± 39.74^*^	296.0 ± 42.22^*^	291.4 ± 43.28^*^

Abbreviations: BCVA, best-corrected visual acuity; logMAR, logarithm of minimal angle of resolution; CRT, central retinal thickness; *P < 0.05 versus baseline.

### 3.3 Comparison of FAZ area, SVD and DVD between groups before and after treatment

**FIGURE 3:**
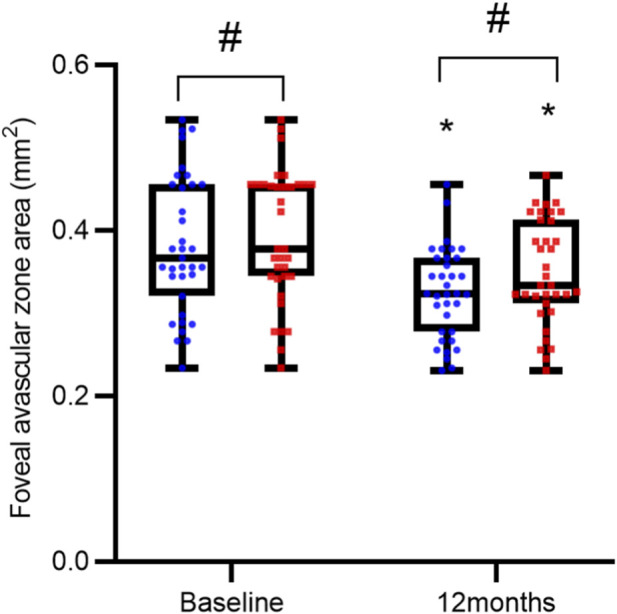
Comparison of Foveal Avascular Zone (FAZ) Area over Time. FAZ measurements are presented as interleaved box-and-whisker plots for the reference ranibizumab (blue) and ranibizumab biosimilar (Rimmyrah, red) groups (n = 35 per group) at baseline, and 12 months. All individual data points are overlaid. Asterisks (*) denote statistically significant reduction from baseline within the same group (P < 0.05). No significant differences were observed between the two treatment groups at any time point (all P > 0.05).

**FIGURE 4:**
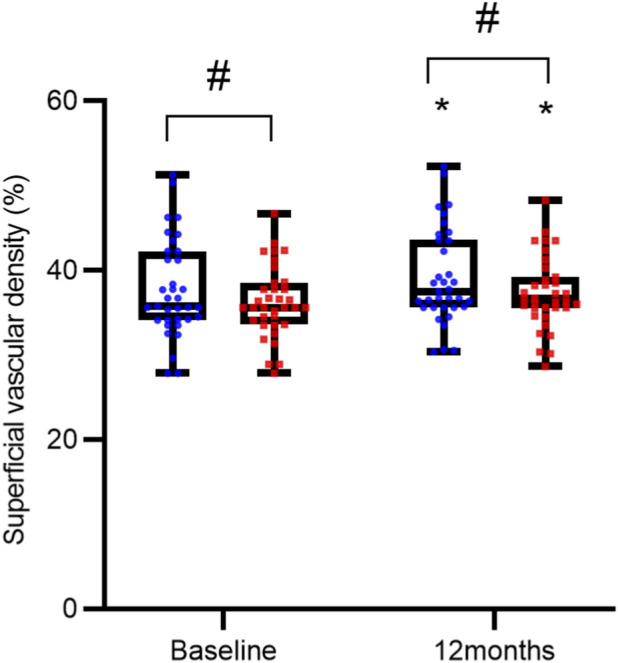
Comparison of Superficial Vascular Density (SVD) over Time. SVD measurements are presented as interleaved box-and-whisker plots for the reference ranibizumab (blue) and ranibizumab biosimilar (Rimmyrah, red) groups (n = 35 per group) at baseline, and 12 months. All individual data points are overlaid. Asterisks (*) denote statistically significant improvement from baseline within the same group (P < 0.05). No significant differences were observed between the two treatment groups at any time point (all P > 0.05).

**FIGURE 5:**
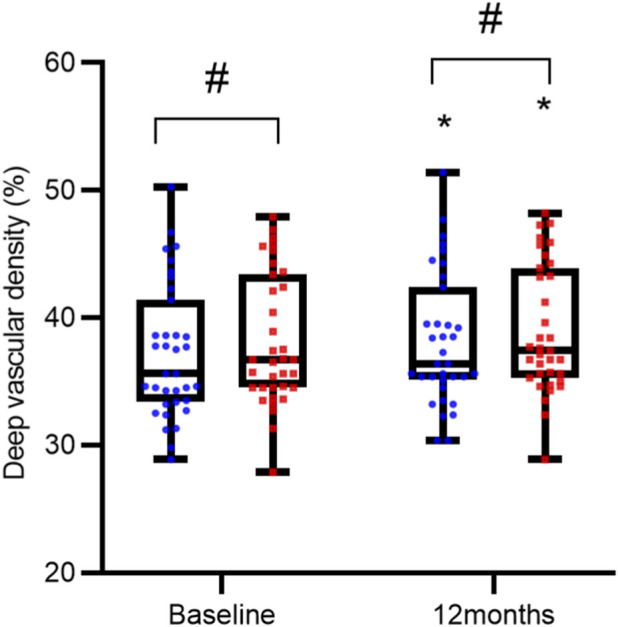
Comparison of Deep Vascular Density (DVD) over Time. DVD measurements are presented as interleaved box-and-whisker plots for the reference ranibizumab (blue) and ranibizumab biosimilar (Rimmyrah, red) groups (n = 35 per group) at baseline, and 12 months. All individual data points are overlaid. Asterisks (*) denote statistically significant improvement from baseline within the same group (P < 0.05). No significant differences were observed between the two treatment groups at any time point (all P > 0.05).

Baseline and post-treatment measurements showed no statistically significant differences between the two groups in terms of FAZ area, SVD, and DVD (all P > 0.05). However, within each group, treatment led to a significant reduction in FAZ area and increases in both SVD and DVD compared to baseline (all P < 0.05), as detailed in Table 3 and Figures 3–5.

**TABLE 3:** Comparison of FAZ area, SVD, and DVD pre- and post-treatment between groups.

Group	Indices (mean ± SD)	Baseline	12 months	P
Reference ranibizumab (n = 35)	FAZ (mm^2^)	0.38 ± 0.08	0.33 ± 0.06	<0.0001
	SVD (%)	37.73 ± 5.76	39.15 ± 5.64	<0.0001
	DVD (%)	37.29 ± 5.25	38.21 ± 5.17	<0.0001
Ranibizumab biosimilar (n = 35)	FAZ (mm^2^)	0.39 ± 0.08	0.35 ± 0.06	<0.0001
	SVD (%)	36.26 ± 4.20	37.29 ± 4.28	<0.0001
	DVD (%)	38.15 ± 5.17	38.99 ± 5.00	<0.0001

Abbreviations: FAZ, foveal avascular zone; SVD, superficial vascular density; DVD, deep vascular density.

### 3.4 Intraocular pressure

The baseline IOP was (16.26 ± 3.08) mmHg in the reference ranibizumab group and (15.20 ± 2.66) mmHg in the biosimilar group, with no statistically significant intergroup difference (P = 0.1287). Following treatment, IOP values were (15.63 ± 2.14) mmHg and (15.40 ± 1.90) mmHg in the reference ranibizumab and biosimilar groups, respectively. Compared with baseline, neither group exhibited a statistically significant change in IOP (P = 0.0736 for the reference ranibizumab group; P = 0.4033 for the biosimilar group).

### 3.5 Comparison of the number of intravitreal injections between the two groups

No statistically significant difference was observed in the mean number of intravitreal injections between the reference ranibizumab group (3.43 ± 0.65) and its biosimilar group (3.69 ± 0.76) during the study period (P = 0.1530), indicating comparable treatment frequency.

### 3.6 Complications

During the 12-month follow-up period, transient intraocular pressure elevation was observed in 1 case and mild corneal epithelial damage in 2 cases in the reference ranibizumab group, while the biosimilar group reported 2 cases of transient intraocular pressure elevation and 1 case of mild corneal epithelial damage. No anterior chamber reaction was recorded in either group. During the 12-month follow-up period, no treatment-related serious ocular adverse events (including cataract, glaucoma, or endophthalmitis) or serious systemic adverse events (such as cardiovascular or cerebrovascular accidents) were observed in either group of patients.

## 4 Discussion

Macular edema represents a significant complication of the microvascular lesions commonly associated with diabetes mellitus. It arises from a series of pathological changes induced by impaired retinal microcirculation. Furthermore, recent studies indicate that the incidence of diabetic retinopathy has been rising annually and has emerged as a leading cause of vision impairment among diabetic patients. VEGF, a potent mitogen and permeability-enhancing cytokine, is primarily secreted by vascular endothelial cells. It functions by stimulating the proliferation, migration, and permeability of nascent endothelial cells, thereby driving pathological neovascularization. In the treatment of DME, anti-VEGF agents are administered to block this signaling pathway, which in turn reduces macular edema (Campochiaro et al., 2014; Wells et al., 2015; Massin et al., 2010). Prior to the advent of anti-VEGF therapy, macular laser photocoagulation was the standard of care for DME. While effective in reducing macular edema, this laser treatment offered limited benefits in terms of visual acuity improvement (Bressler et al., 2016).

The current anti-VEGF armamentarium for diabetic macular edema (DME) comprises conbercept, aflibercept, and ranibizumab (Lin et al., 2025; Liu and Li, 2019; Jiajia et al., 2024; Zhou et al., 2021; Liberski et al., 2022; Kun et al., 2021). The combined effect of patent expirations for originator biologics and advances in biotechnology has spurred the development of biosimilars. These products have well-established quality, safety, and efficacy profiles that are comparable to their reference products (Ashish et al., 2025). OCTA is a rapid, non-invasive imaging technology that has emerged recently. It enables the quantitative assessment of retinal capillary vessel density and foveal avascular zone area in the macular region. The effect of anti-VEGF therapy on macular perfusion remains controversial. Some studies (Campochiaro et al., 2014) suggest it improves perfusion, whereas others indicate it may exacerbate retinal ischemia (Shimura and Yasuda, 2010). Previous studies have reported that the biosimilar ranibizumab Razumab© can serve as an effective treatment option for wet age-related macular degeneration, diabetic macular edema, and retinal vein occlusion, demonstrating short-term efficacy by reducing central foveal thickness and improving visual acuity (Gopal et al., 2020).

To our knowledge, there are no published reports from clinical studies directly comparing the efficacy of a ranibizumab biosimilar (Rimmyrah) and reference ranibizumab in patients with DME. This study aimed to evaluate the differences in therapeutic efficacy between a ranibizumab biosimilar and the reference ranibizumab in patients with DME by comparing BCVA, CRT, FAZ area, SVD and DVD in the macular region, as well as the incidence of complications before and after treatment.

The results of this study showed that both groups had significant improvements from baseline in BCVA and CRT at 3, 6, and 12 months post-treatment (P < 0.05). At the 12-month follow-up, FAZ area was significantly reduced, while both the SVD and DVD were significantly increased compared to pre-treatment values (P < 0.05). This is consistent with the results of previous studies (Campochiaro et al., 2014). However, there were no statistically significant differences in any outcome measures between the two groups at any time point. The main risks associated with intravitreal anti-VEGF therapy include infection, hemorrhage, glaucoma, cataract, and cardio-cerebrovascular accidents (Schmidt-Erfurth et al., 2014). Elevation of intraocular pressure is among the most commonly reported adverse events. Compared with baseline, neither group exhibited a statistically significant change in IOP. During the study period, no other systemic or serious ocular safety concerns were identified. Over a 12-month follow-up, this study provides evidence that the ranibizumab biosimilar (Rimmyrah) showed similar safety and efficacy profiles to its reference product in the treatment of DME. This conclusion is consistent with previous research findings on other ranibizumab biosimilars. For example, a Phase III randomized controlled trial comparing OPTIMAB® (another ranibizumab biosimilar) with the innovator product demonstrated that there were no statistically significant differences between the two in terms of visual acuity benefits, anatomical improvements, and safety in the treatment of patients with neovascular age-related macular degeneration (AMD), establishing the non-inferiority of the biosimilar (Parth et al., 2024). Extending the evaluation to the microvasculature using OCTA, we found that the two treatments yielded similar improvements in macular perfusion parameters, such as FAZ area, SVD, and DVD. These results bridge evidence from conventional efficacy measures to microvascular restoration, thereby reinforcing the biosimilar’s holistic therapeutic value in managing DME.

Although improvements in the FAZ area and vascular density were observed, caution is warranted in directly equating these OCTA findings with true microvascular reperfusion, due to known methodological constraints such as segmentation errors and projection artifacts. The OCT-A improvements noted in our study should thus be regarded as a promising anatomical correlate, providing preliminary support for similar microvascular effects between the two agents. Their definitive clinical significance, however, requires further validation alongside functional visual outcomes.

The results are consistent with the scientific basis for biosimilar approval, which requires high similarity in amino acid sequence, three-dimensional structure, target affinity, and pharmacological mechanism to the reference product. With comparable efficacy established, the principal advantage of the biosimilar lies in its substantially lower cost. Given that treatment expense significantly influences patient adherence, reducing the financial burden may improve treatment persistence and long-term outcomes. In an era increasingly oriented toward value-based healthcare, biosimilars represent a viable strategy for delivering equivalent clinical benefits at a lower cost.

While the relatively conservative “3+PRN” regimen used in this study (with fewer mean annual injections) may reduce treatment burden, it could also explain the more modest magnitude of visual improvement observed compared to some intensive treatment protocols. Nevertheless, the highly similar therapeutic responses and outcomes exhibited by both groups under this regimen precisely underscore the comparability of the biosimilar in real-world clinical practice.

This study thus offers evidence from both efficacy and economic viewpoints to inform clinical practice: choosing a biosimilar without sacrificing effectiveness can optimize healthcare resource allocation and align with public health priorities. Several limitations should be noted, however, including the modest sample size, the 12-month follow-up duration, and the absence of a formal cost-effectiveness analysis (CEA). Moreover, the limitations of this study include its retrospective design and the relatively small sample size (35 patients per group), which may have limited the statistical power and increased the risk of a Type II error, thereby reducing the ability to detect potential minor differences between the two groups. Future real-world investigations with larger cohorts and extended observation periods are warranted to confirm long-term efficacy and safety. Further health-economic studies are also needed to quantify the cost savings and health gains associated with biosimilar use in DME, thereby providing stronger evidence for policy-making.

In conclusion, the ranibizumab biosimilar represents an effective and cost-efficient treatment option for DME. We recommend that clinicians consider efficacy, safety, patient financial status, and local healthcare resource availability when selecting therapy, and regard biosimilars as a rational alternative in management plans. Ongoing surveillance of real-world performance and safety, coupled with clear patient-clinician communication regarding treatment options, will support optimized outcomes in DME care.

## Data Availability

The datasets used or analyzed during the current study are available from the corresponding author on reasonable request.
